# Recent advances in corneal specular microscopy image analysis through artificial intelligence

**DOI:** 10.1371/journal.pdig.0001305

**Published:** 2026-03-31

**Authors:** Andres G. Marrugo, Fernando Quintero, Alejandro Tello, Angélica M. Prada, Virgilio Galvis, Lenny A. Romero

**Affiliations:** 1 Escuela de Ingeniería, Arquitectura y Diseño, Universidad Tecnologica de Bolivar, Cartagena, Colombia; 2 Centro Oftalmológico Virgilio Galvis, Floridablanca, Colombia; 3 Fundación Oftalmológica de Santander FOSCAL, Floridablanca, Colombia; 4 Faculty of Health, Universidad Autónoma de Bucaramanga UNAB, Bucaramanga, Colombia; 5 School of Medicine, Faculty of Health, Universidad Industrial de Santander UIS, Bucaramanga, Colombia; 6 Dirección de Ciencias Básicas, Universidad Tecnologica de Bolivar, Cartagena, Colombia; University College London Institute of Ophthalmology, UNITED KINGDOM OF GREAT BRITAIN AND NORTHERN IRELAND

## Abstract

Although conventional automated analysis of corneal specular microscopy images has historically been limited by reproducibility challenges in the presence of corneal guttae, recent advances in artificial intelligence (AI) have significantly enhanced its diagnostic potential in such cases. This review explores the integration of AI techniques for analyzing specular microscopy images, emphasizing the shift from classical to advanced AI methods. We highlight AI-based methodologies—supervised and unsupervised learning—that have significantly enhanced the accuracy of *in vivo* human corneal endothelium analysis. The paper also discusses the challenges in data collection, emphasizing ethical considerations and the need for high-quality datasets. Additionally, we explore novel AI-derived metrics and their implications in enhancing diagnostic precision, particularly in Fuchs endothelial corneal dystrophy. The review concludes with insights into the future direction of AI in specular microscopy, highlighting its increasing relevance in ocular healthcare and the potential to overcome longstanding limitations in the field.

## Introduction

**Fig 1:**
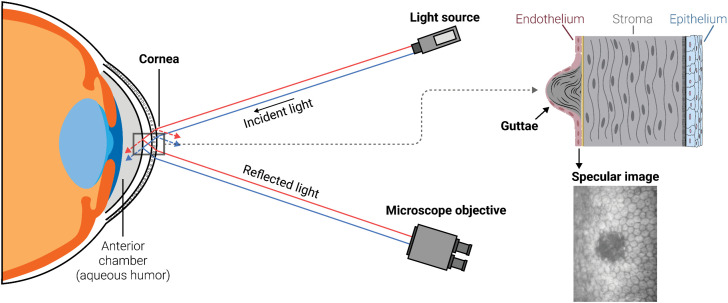
Optical principle of specular microscopy. The device comprises a light source and a microscope coupled to a camera (left). When reflected light from the corneal endothelium/aqueous humor interface aligns with the microscope’s optical axis, the endothelium becomes visible. In cases of Fuchs’ dystrophy, the endothelium images display characteristic dark spots known as guttae (right).

For over half a century, the crucial role of human corneal endothelial (CE) cells in maintaining corneal transparency and regulating stromal hydration has been well-established. These cells, through both passive and active mechanisms, maintain stromal hydration within a strict range (approximately 78%). Interestingly, in humans they also exhibit a near-complete inhibition of *in vivo* mitosis [1,2]. Specular microscopy enables in vivo imaging of the CE and plays a vital role in extracting valuable clinical data. Currently, using classical image processing methods, it allows for segmentation of individual cells to derive crucial morphometric parameters such as cell density, mean cell area, hexagonality, and coefficient of variation of cell area (Fig 1) [3,4].

**Fig 2:**
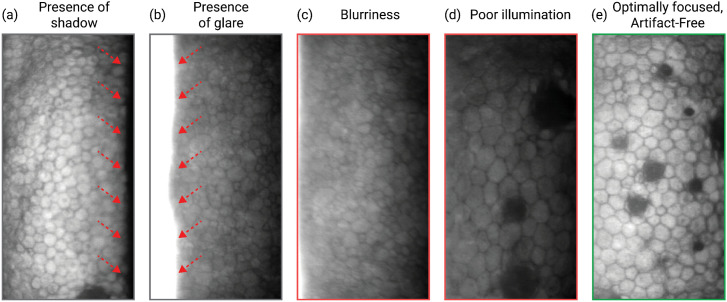
Series of specular microscopy images showcasing common acquisition challenges and a high-quality capture. Images (a) through (d) demonstrate various issues encountered during image acquisition, including shadows, glares, blur, and poor illumination, which can impede accurate analysis. Image (e) represents an ideal scenario: a perfectly focused and artifact-free image, capturing a cornea with guttata. This contrast highlights the importance of image quality for reliable specular microscopy analysis.

Despite being a valuable tool, modern specular microscopy still faces significant limitations, particularly in cases of Fuchs endothelial corneal dystrophy (FECD). This condition is characterized by the presence of guttae, abnormal excrescences of Descemet membrane (DM) that obscure visualization of CE cells. In FECD, the accurate identification of healthy endothelial cells and the detection of their boundaries are challenging, impacting the analysis of cell density and other morphometric parameters [3,5–7]. Corneal edema, often present in advanced stages of FECD, further complicates the acquisition of adequate images, as depicted in Fig 2. This figure illustrates common acquisition challenges in specular microscopy images, such as shadows, glares, blur, and poor illumination, which impede accurate analysis. Image (e) in the figure represents an ideal scenario with a perfectly focused and artifact-free image, capturing a cornea with guttae, emphasizing the importance of image quality for reliable specular microscopy analysis. While *in vivo* confocal microscopy has shown better performance in some cases, its widespread adoption is hindered by cost and technical requirements [8–10].

The importance of FECD lies in its clear connection with corneal edema and the detrimental effect of guttae on human CE cells (HCECs). Studies have shown a link between *in vivo* HCECs density reduction and the presence of DM excrescences in FECD, and larger guttae have been found to adversely affect HCECs growth [11–14]. Given the ongoing research into non-transplant interventions for FECD, it is crucial to understand HCECs density and morphology, and how these correlate with the presence of guttae [2,12,14–20].

To address these issues, manual corrective approaches have been developed to determine the Effective Endothelial Cells Density (EECD) and calculate the percentage of areas covered by guttae [9,21]. However, in recent years, artificial intelligence (AI) has emerged as a powerful tool for CE specular microscope image analysis, enabling more reliable morphometric measurements even in the presence of FECD [22–26]. This review examines the recent advances in AI technologies for the processing and segmentation of specular corneal microscopy images, highlighting how these new AI-based image analysis tools render specular microscopy a valuable tool for assessing the health of the CE in FECD and similar conditions.

### Ethics statement

Data used in this review paper are from the project “New classification of specular microscopy images of the corneal endothelium in Fuchs dystrophy through artificial intelligence techniques” (Project code 763–2021), approved by the ethics committee of the Universidad Tecnológica de Bolívar, Colombia. The review utilizes this data solely for illustrative purposes. Informed consent was obtained for all original project participants, and the data, anonymized for privacy, is available at the Open Science Framework repository (https://osf.io/75kmu/), https://doi.org/10.17605/OSF.IO/75KMU.

## Classical methods in specular microscopy

The automated analysis of specular microscopy images in commercially available devices has traditionally relied on classical image processing methods, and this remains the case today. The recent progress in artificial intelligence is not yet readily available for clinical use [27]. Techniques like contour detection, mathematical morphology, and Fourier analysis continue to be applied for extracting information from specular images, providing essential data on cell size and morphology for diagnosing various corneal conditions. One of the most commonly implemented algorithms for specular microscopy images is the watershed transform, often combined with various filters and grayscale morphological operations to segment cells [28–30]. While these techniques effectively segment healthy corneal endothelium, their application in FECD cases remains challenging. This challenge is primarily due to the unique morphological changes caused by the presence of guttae.

Numerous researchers have proposed alternative methods for automated analysis. For instance, Scarpa and Ruggeri [31] introduced a method following a conventional image processing approach. It involves the initial detection of cell centers using Laplacian of Gaussians, followed by the derivation of cell contours with Euclidean skeletons, and ultimately refining these contours through genetic algorithms. The outcomes were optimal, and the morphometric parameters obtained were in agreement with manual segmentation reference values. However, it is important to note that the study was conducted using images from corneas without guttae. Extending this approach to images with abnormalities (such as FECD or other endothelial dystrophies) is not readily feasible and may require significant fine-tuning and heuristics. The critical issue is that these contour-based methods are not designed to cope with abnormal corneal features like guttae, which are supposed neither to be considered as cells nor simply disregarded, as this leads to under- or over-estimation of crucial parameters, such as endothelial cell density

Karmakar et al. [32] developed an automatic image analysis algorithm for corneal endothelial cells, utilizing classical techniques. To address challenges like poor image quality, they employed multi-step filtering, including Gaussian blur and Laplacian of Gaussian edge detection, to enhance cell boundary detection. Morphological operations were used to handle uneven illumination and cell over-segmentation. The algorithm iteratively applied these steps, with increasing standard deviations, and averaged the results. Finally, they applied stochastic watershed segmentation on the image. The calculated parameters included cell density, hexagonality, and coefficient variance. They excluded guttae of endothelial images from patients with FECD from analysis, by masking out them when implementing a Region of Interest demarcation based on a local entropy filter. They compared the results with some recent works with AI-methods showing high accuracy computing morphometric parameters. Despite the algorithm’s precision, its complexity introduces a trade-off in computational efficiency and in addition, it has important limitations when applied to FECD, since excluding guttae from the region of interest leads to inaccurate cell density determinations, causing an overestimation and failing to provide meaningful information in such cases.

Specular microscopes, in addition to manual identification based methods for semi-automatic processes, often feature proprietary software that perform semi-automatic or automatic analysis of endothelial cells by executing cell segmentation and calculating morphometric parameters like cell density, area, and shape [28–30,33]. Although the automated approaches speed up the analysis compared to manual methods and reduces subjectivity, they can make mistakes in cases of low-quality images [34].

**Fig 3:**
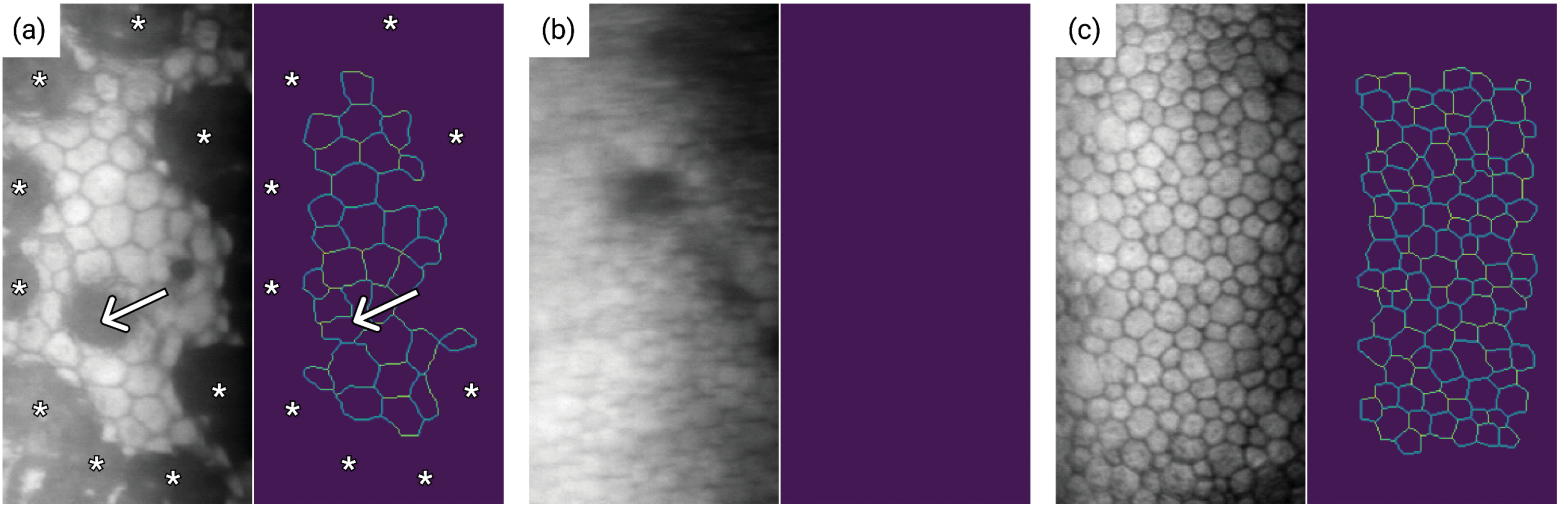
Comparative outcomes of cell segmentation with classical methods by built-in software in a specular microscope (Topcon SP-3000P): (a) Result of inaccurate segmentation in endothelium affected by Fuchs’ endothelial corneal dystrophy. Guttae are either excluded from detection and analysis (asterisks) or wrongly segmented as containing healthy cells (arrowhead). **(b)** No segmentation due to a blurred image. **(c)** Correct segmentation in a healthy corneal endothelium.

In addition, the automated programs included in the devices are much less accurate with images from diseased corneas (e.g., in the presence of corneal guttae) like in FECD. In Fig 3(a), the segmentation output from the Topcon SP3000 (Topcon, Tokyo, Japan) software on a corneal endothelium affected by FECD is shown. Here, the software’s limitations in accurately identifying and segmenting guttae are evident. Fig 3(b) presents a different challenge where the image’s blurriness impedes the software’s ability to effectively identify cells. In contrast, Fig 3(c) demonstrates a successful segmentation, due to the corneal endothelium image being free from guttae and blur.

## Advances in AI-based methods

In recent years, artificial intelligence (AI) has revolutionized specular microscopy, offering groundbreaking methods for analyzing corneal endothelium images. This section explores this revolution from foundational AI techniques to sophisticated automated segmentation and cutting-edge innovations. We highlight how these developments have enhanced the precision and efficiency of corneal image analysis, marking a significant leap in corneal health assessment.

### Foundations of AI in Specular Microscopy

AI encompasses methods capable of learning from inputs and executing complex tasks. In the context of specular microscopy, AI refers to the automatic assessment of corneal endothelium images through machine learning techniques. These methods require data to learn the segmentation task, crucial for computing accurate morphometric parameters.

Lopes et al. [35] provided an insightful overview of AI applications in corneal diagnosis, emphasizing the evolution of AI techniques in managing a wealth of data from multimodal imaging devices in corneal diseases, and a broader survey that spans the full spectrum of corneal diseases has since been published [36]. This underscores the growing importance and potential of AI in enhancing clinical decisions, especially in the field of corneal health.

AI-based methods have been used in ophthalmology with notable success, particularly across a range of anterior-segment disorders [37–39], and continue to evolve as larger annotated datasets become available. Traditionally, AI in this field has relied on image processing pipelines augmented with manual feature engineering and machine learning techniques, such as classification trees and neural networks [40]. Foracchia et al. [41] discussed early approaches in corneal endothelium cell field analysis, highlighting the limitations of these methods, particularly their performance in varying image quality levels and challenging conditions like FECD. This sets the stage for the need for advanced AI methods that are more adaptable to the variability in specular images and can handle complex cases more effectively.

**Fig 4:**
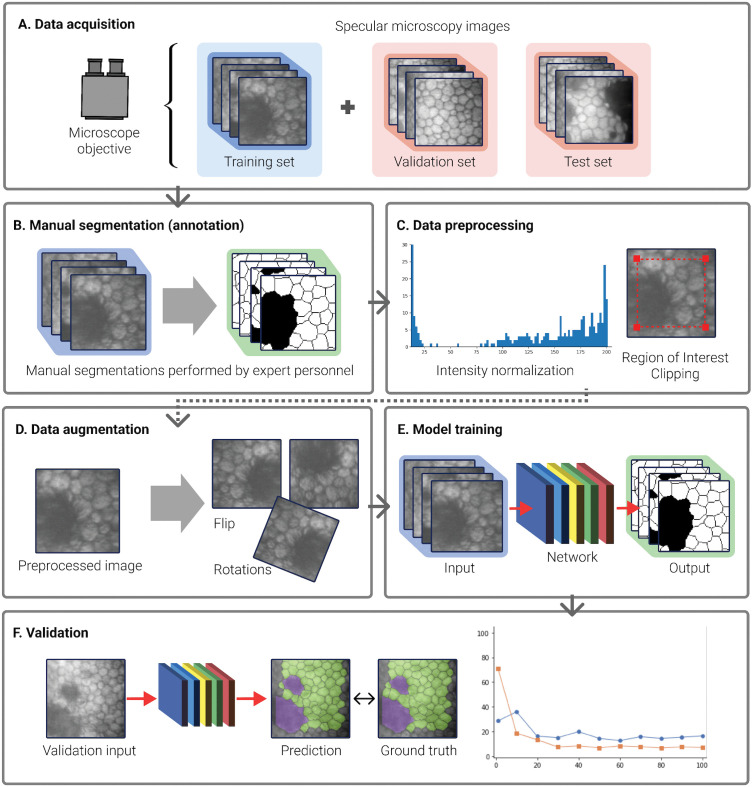
Workflow of AI-based Methods in Specular Microscopy. **(a)** Division of data into training, validation, and test sets. **(b)** Manual segmentation and annotation by specialists. **(c)** Preprocessing stages including intensity normalization and illumination correction. **(d)** Data augmentation techniques. **(e)** Training phase until convergence. **(f)** Validation testing to ensure model generalization.

The efficacy of AI in medical imaging arises from its ability to process numerous examples covering a wide range of scenarios. This enables the algorithm to learn the segmentation task from data representative of real-world challenges. The data acquisition process involves dividing the dataset into training, validation, and test sets (Fig 4(a)). The training set is used to teach the AI model, the validation set helps in tuning the parameters and preventing overfitting, and the test set is used to evaluate the model’s performance [42]. Specialists manually segment and annotate the images (Fig 4(b)), followed by preprocessing steps like intensity normalization and non-uniform illumination correction (Fig 4(c)). Data augmentation techniques such as rotations and flips (Fig 4(d)) enhance the neural network’s generalization capabilities. The training phase continues until convergence or a defined stopping criterion is met (Fig 4(e)). Validation tests are conducted during training to ensure effective generalization (Fig 4(f)). Upon successful training and validation, the network is ready for practical application.

### Automated segmentation and enhanced accuracy

The implementation of convolutional neural network (CNN) architectures, particularly the U-Net, has significantly advanced automated segmentation in specular microscopy. The U-Net model, originally introduced by Ronneberger et al. [43], has been a cornerstone in biomedical image segmentation due to its effectiveness in various image qualities. Daniel et al. [44] extended the application of this architecture to segment corneal endothelial cells (CECs) in a wide range of specular microscopy images, showcasing its robustness in varying conditions, including those with guttae and stromal edema.

To address the specific challenges posed by FECD, Shilpashree et al. [45] adapted the U-Net architecture, demonstrating high accuracy in segmenting corneal endothelial cells in FECD. This disease, prevalent in older populations and more common in women, leads to the accumulation of guttae, which appear as dark regions in specular microscopy images [46, 47]. Accurate segmentation in such conditions is crucial for effective disease management and progression follow-up.

Vigueras-Guillén et al. explored different enhancements to the U-Net architecture. They initially utilized a fully convolutional architecture based on U-Net, along with a sliding-window CNN, for assessing CE images [48, 49]. Additionally, they implemented a densely connected U-Net architecture for identifying the region of interest (ROI) in specular microscopy images [50]. Their subsequent work included a CNN-based regression to estimate biomarkers in these images, combining various methods for improved accuracy [51]. Notably, in their recent study, they introduced DenseUNets with a feedback non-local attention mechanism to enhance segmentation accuracy in CE images with guttae [25]. This approach, however, required extensive manual segmentation and sophisticated post-processing techniques.

Recent AI-based methods, like those of Vigueras et al. [25], have adopted a segmented approach to CE image analysis: a CNN to deduce cell edges, another model for identifying full cells, and an image processing technique based on watershed for refining edges, selecting well-detected cells, and extracting corneal parameters (postprocessing). This multi-faceted approach is crucial, as output often presents issues such as discontinuity and isolated cell boundaries, where watershed transformation becomes indispensable. Similar strategies are employed by Kucharski et al. [52] and Sierra et al. [53], who segment the CE into multiple classes, including cell borders, edges, and centers. Sierra et al. [53] further distinguish guttae as a separate class. Postprocessing with watershed transform is applied to ensure reliable segmentation, addressing the variability in corneal endothelium images.

These advancements in AI-driven automated segmentation, especially through the adaptation and enhancement of the U-Net architecture, represent a significant leap in the application of specular microscopy. They offer improved accuracy and adaptability, essential for dealing with diverse clinical conditions and image qualities in corneal health assessment.

In Table 1 we provide a structured comparison of recent studies employing automated methods for corneal endothelial analysis. For each study, we summarize the network architecture, dataset size (number of images and subjects), study population, reported performance metrics, use of data augmentation, and whether guttae were modeled as a separate semantic class. By consolidating these parameters, the table enables direct comparison of methodological design choices, dataset scale, and evaluation strategies across investigations.

**Table 1:** Summary of studies in automated corneal endothelial analysis.

Study	Network	Images^1^	Sample^2^	Population	Performance Metrics^3^	Data Aug.	Guttae Class^4^
Daniel et al. [44]	U-Net	385	385	Healthy, FECD, Post-EK	PCC *R*^2^: 0.96; PPV: 84%		
Shilpashree et al. [45]	Mod. U-Net + Watershed	246	130	Healthy, FECD	Accuracy: 87.9%; AUC: 0.967	✓	✓
Kucharski et al. [52]	Mod. U-Net + Watershed	119	–	Animal, Healthy, FECD	Dice: 0.868; PCC *R*^2^: 0.929		
Vigueras et al. [25]	Dense U-Net	1203	–	Healthy, FECD	MAE ECD: 23.16 cells/mm^2^	✓	✓
Sierra et al. [53]	UNet-dm (Regr.)	90	66	Healthy, FECD	Accuracy: 83.79%	✓	✓
Foo et al. [54]	DenseNet-121	775	637	Healthy, FECD	AUC: 0.96; Sensitivity: 0.91		✓
Sanchez et al. [55]	Semi-sup. CNN	90	66	Healthy, FECD	Dice: 90.1%; IoU: 82.1%	✓	✓
Tey et al. [56]	U-Net	2193	50	Healthy, FECD	Dice: 0.86 ± 0.04 (cells) / 0.90 ± 0.04 (guttae); PCC (r): 0.91 (ECD)	✓	✓
Qu et al. [57]	ECCT	4172	532	Healthy, FECD, Viral, PPCD, ICE	Accuracy: 89.53%; AUC: 0.958	✓	✓

^1^Quantity prior to data augmentation.

^2^Quantity of persons (human subjects).

^3^Reported metrics vary across studies and are influenced by the specific size and characteristics of the validation/test datasets.

^4^Indicates whether the model identifies and segments guttae as a separate semantic class.

✓ = Feature addressed. PCC = Pearson Correlation. PPV = Positive Predictive Value. MAE = Mean Absolute Error. IoU = Intersection over Union.

The studies included exhibit substantial heterogeneity in both dataset size and clinical composition. For example, Tey et al. [56] reported Dice coefficients of 0.86 for cells and 0.90 for guttae using a U-Net architecture trained on 2193 images from 50 subjects, whereas Qu et al. [57] leveraged a larger and more clinically diverse dataset (4172 images from 532 subjects) spanning healthy, FECD, viral keratitis, PPCD, and ICE populations, achieving an accuracy of 89.53% and an AUC of 0.958. Such differences underscore the importance of interpreting reported metrics in light of dataset composition and validation design.

Overall, the comparison suggests that contemporary convolutional architectures—when adequately trained—tend to achieve broadly comparable segmentation or classification performance. As illustrated by Sanchez et al. [55], performance gains may arise less from architectural novelty and more from strategies such as data augmentation, semi-supervised learning, and improved label representation. Consequently, dataset representativeness, annotation quality, and evaluation protocols appear to be critical determinants of robustness and clinical applicability, rather than network design alone.

### Innovative techniques

In pursuit of enhancing AI-based methods in specular microscopy, researchers have explored innovative techniques to overcome the limitations of traditional approaches. Manual segmentation of thousands of images is a laborious task, spurring interest in self-supervised and semi-supervised learning methods. Self-supervised learning involves training models using automatically generated labels or intrinsic properties of the data, thus reducing the need for manually annotated datasets. Semi-supervised learning, on the other hand, uses a small amount of labeled data along with a larger set of unlabeled data, making it a practical approach in scenarios with limited annotated images.

Sanchez et al. [55] demonstrated the potential of self-supervised learning in specular microscopy. Their approach, focused on enhancing the encoder of a UNet model, showed accurate segmentation performance even with reduced training datasets. This advancement is crucial in contexts where obtaining extensive annotated data is challenging. Building on this line of work, a subsequent study [58] proposed a warping-based data augmentation technique to model natural variability in the corneal endothelium, which further improved semi-supervised segmentation performance across multiple CNN architectures.

Another frontier in AI research involves the use of Generative Adversarial Networks (GANs) for expanding training data synthetically. Kucharski et al. [22] investigated this aspect by generating synthetic images of the corneal endothelium, raising questions about the necessity of expert annotation in the training process. However, Mendoza et al. [59] found that training GANs can be challenging, as the network might learn noise, affecting the training of subsequent models.

Beyond segmentation, AI applications are extending to predictive and prognostic analysis. Joseph et al. [60] demonstrated that machine learning models trained on postkeratoplasty endothelial cell images can predict future graft rejection events before clinical decompensation becomes apparent. In their study, texture and morphometric features extracted from specular microscopy images were used to train predictive classifiers, achieving robust discrimination performance, with area under the receiver operating characteristic curve (AUC) values exceeding 0.80. This work highlights the potential of AI not merely to quantify endothelial morphology, but to identify subtle patterns associated with impending graft failure, thereby enabling earlier clinical intervention.

Similarly, Foo et al. [54] showed that deep learning models applied directly to widefield specular microscopy images can detect FECD without requiring prior cell segmentation. Their segmentation-free approach achieved high diagnostic performance, underscoring that predictive modeling may leverage global image patterns beyond conventional morphometric descriptors. The ability to recognize disease states directly from raw imaging data suggests that AI systems can capture latent structural and textural signatures that are not readily quantified through traditional density-based metrics.

Together, these studies reflect a paradigm shift from descriptive morphometry toward risk stratification and outcome prediction. Rather than focusing exclusively on static measurements, emerging AI frameworks aim to forecast clinically meaningful endpoints—including graft rejection and disease progression—supporting a more proactive and preventative model of corneal care.

**Fig 5:**
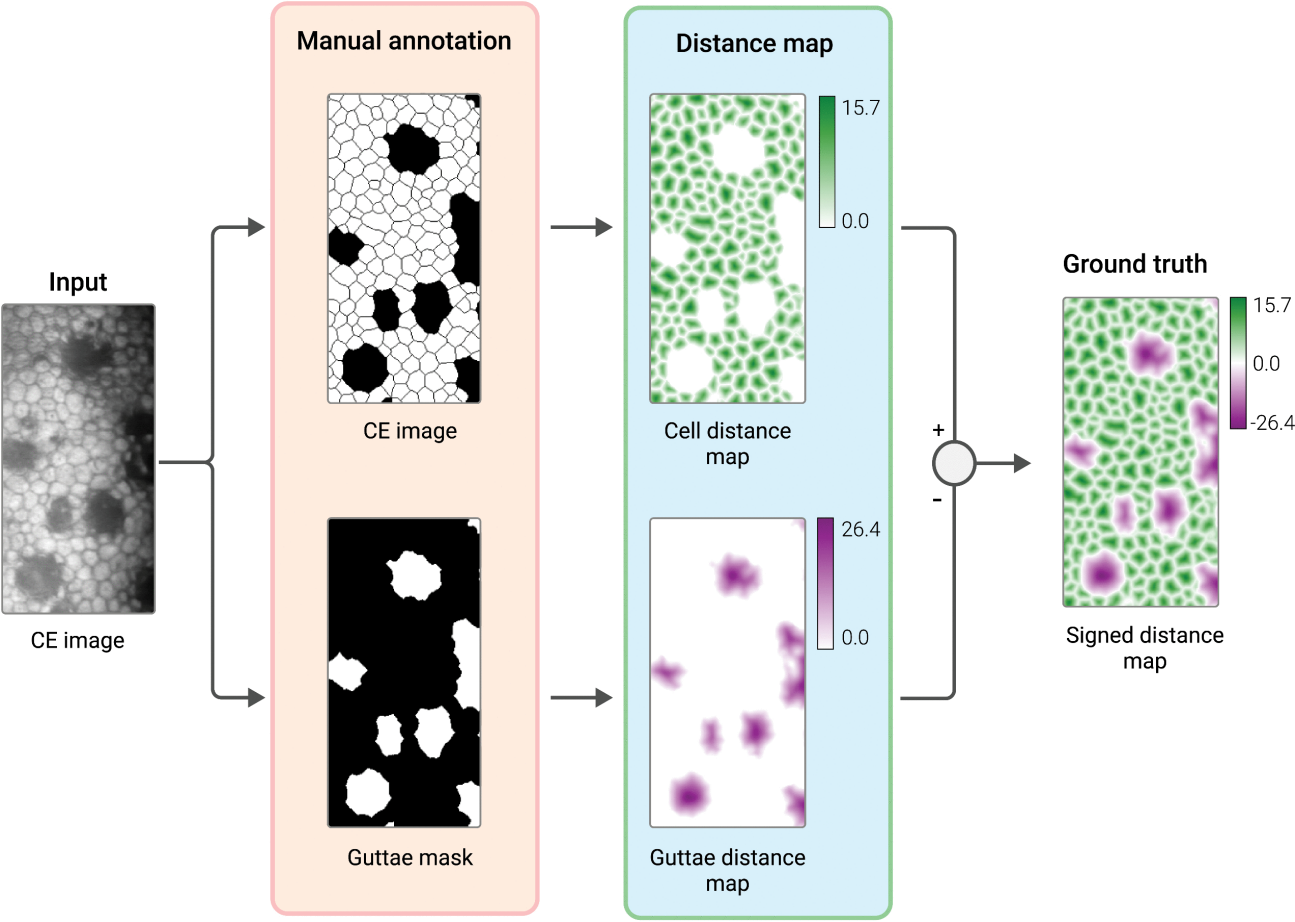
Signed distance maps in cell and guttae segmentation. This figure illustrates the approach by Sierra et al. [24] where manual segmentations of corneal endothelial cells and guttae are first generated. These segmentations are then transformed into signed distance maps, depicting a smooth transition from the centers to the boundaries of cells (positive values) and guttae (negative values). The maps facilitate the training of the network and the comparison of predicted maps against the groundtruth. [Adapted] with permission from [24] © The Optical Society.

Sierra et al. [24] introduced a novel deep regression method to model cells and guttae in specular microscopy images as signed distance maps, effectively differentiating between healthy corneal endothelial cells and guttae-affected areas. This method allows for the calculation of EECD and guttae area ratio (GAR%), crucial for accurately characterizing corneal endothelium stages. Recognizing the challenges in manual segmentation accuracy, their approach treats manual delineations as fuzzy approximations rather than exact groundtruth. They convert manual segmentations into signed distance maps [61], providing a smooth transition from cell or guttae centers to the boundaries (Fig 5). A U-net-like architecture is then employed to regress the input images to these distance maps, using mean squared error as a loss metric for training instead of classification error from segmentation masks. This method, first of its kind for simultaneous segmentation of cells and guttae, builds upon previous uses of distance maps in corneal endothelium cell segmentation [62].

A key implication of this paradigm is that EECD and GAR% extend beyond traditional endothelial cell density (ECD) measurements obtained from commercial built-in software, which frequently exclude guttae-covered areas from analysis. By incorporating the total analyzed area—including regions occupied by guttae—EECD avoids the systematic overestimation of cell density commonly observed in FECD. GAR%, in turn, quantifies the proportion of the analyzed area occupied by guttae and serves as a structural marker of endothelial compromise.

The clinical relevance of these AI-derived morphometric parameters has been validated by Prada et al. [26], who compared EECD, GAR%, hexagonality, and coefficient of variation against the modified Krachmer (m-Krachmer) clinical grading system. In the central cornea, GAR% demonstrated the strongest correlation with disease severity (*r* = 0.60, *P* < 0.001), outperforming conventional ECD measurements. Importantly, their study showed that standard automated microscope software significantly overestimates endothelial cell density in FECD cases (mean 2216 cells/mm^2^) compared with AI-derived EECD (mean 1322 cells/mm^2^), primarily because guttae-affected regions are excluded from conventional calculations. In multivariable analysis, GAR% emerged as the only parameter independently associated with m-Krachmer grade, underscoring its value as a clinically meaningful biomarker of FECD severity.

From a clinical perspective, these findings support several practical applications. First, GAR% provides an objective and reproducible indicator of endothelial structural dysfunction, particularly in early and intermediate FECD stages where conventional ECD values may remain deceptively reassuring. Second, because FECD progression and postoperative corneal decompensation are closely related to endothelial functional reserve, GAR% and EECD may assist in stratifying the risk of irreversible corneal edema following phacoemulsification in FECD patients [26]. Third, these metrics offer a quantitative framework for monitoring emerging regenerative endothelial therapies, such as Rho-associated protein kinase (ROCK) inhibitor–based treatments, in which reductions in guttae burden or improvements in effective cellular coverage may precede detectable changes in conventional density measurements.

**Fig 6:**
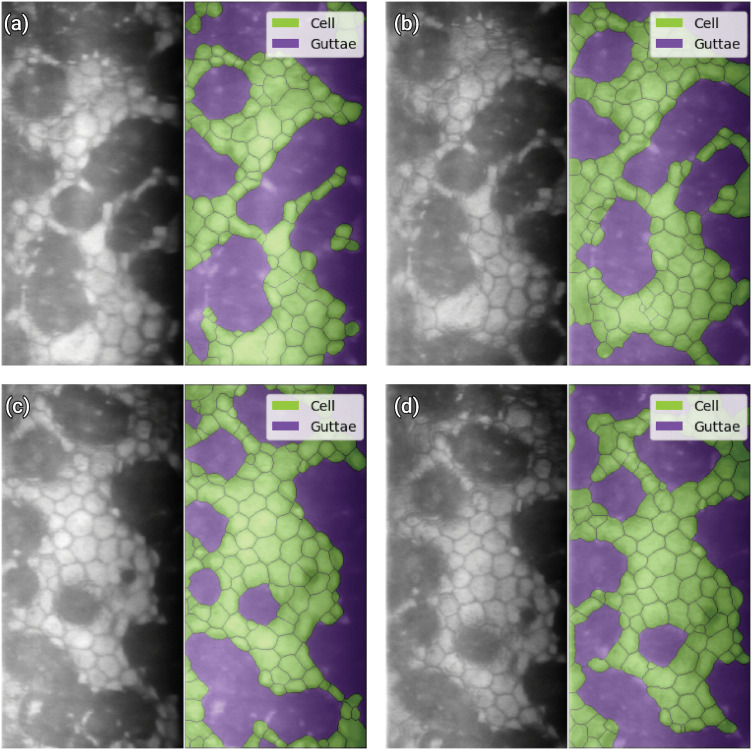
Variability in guttae distribution in specular microscopy acquisitions, illustrating the method by Prada et al. [26]. Images (a) to (d) show the four acquisitions with respective AI-based segmentation and computed EECD values of (a) 1100 cells/mm^2^, (b) 1426 cells/mm^2^, (c) 1337 cells/mm^2^, and (d) 1296 cells/mm^2^. Noticeable shifts in guttae positioning across acquisitions lead to varying morphometric parameters, highlighting the need for using the median value as a robust measure for assessing corneal endothelium health.

To enhance robustness and clinical reliability, Prada et al. [26] further proposed computing morphometric parameters from multiple acquisitions of the same corneal region and reporting the median value. This strategy mitigates local variability in guttae distribution and reduces the influence of focal artifacts, thereby improving repeatability and strengthening the translational potential of AI-based specular microscopy in routine clinical practice (Fig 6).

These innovative techniques emphasize a paradigm shift in AI-based specular microscopy, moving towards more efficient, accurate, and predictive models, with the potential to revolutionize corneal health assessment and treatment.

## Current challenges and limitations of AI-based methods

While AI-based methods in specular microscopy have shown remarkable potential, their translation into clinical practice faces significant challenges [63]. These challenges span various aspects, from data limitations to concerns about the generalizability [64] and longevity of AI systems. Recent work has also highlighted how biases embedded in AI algorithms can amplify health disparities if left unchecked [65].

### Data limitations and dataset representativeness

A primary challenge in developing effective AI systems for specular microscopy is the limited availability of sufficiently large and clinically diverse datasets. Robust model development requires data that capture the variability encountered in real-world settings, including differences in disease severity, image quality, acquisition devices, and patient demographics. While machine learning research often emphasizes the importance of “balanced” datasets, strict class balance—where all diagnostic categories are equally represented—does not reflect clinical reality. In practice, healthy and early-stage cases are far more common than advanced or rare conditions. Rather than artificially equalizing class frequencies, datasets should aim to be representative of real-world prevalence, while appropriate statistical strategies (e.g., class weighting or targeted augmentation) may be used during training to mitigate imbalance without distorting evaluation. Importantly, while imbalance can be addressed during model training, performance should be evaluated on datasets that reflect real-world prevalence to avoid overly optimistic estimates of clinical utility.

Federated learning has been proposed as a potential strategy to address data limitations by enabling collaborative model training across institutions without centralized data pooling [66]. This approach may facilitate the development of larger and more diverse datasets while adhering to privacy and ethical standards. However, its advantages in specular microscopy should be interpreted with caution. When aggregating data from multiple sites, models may inadvertently learn “shortcut” features associated with institution-specific acquisition characteristics—such as illumination settings, device calibration, or preprocessing pipelines—rather than pathology-specific patterns. If certain centers disproportionately contribute specific disease stages, the model may associate site-related signatures with disease labels, thereby compromising generalizability.

Moreover, the degree to which specular microscopy images raise privacy concerns remains an open question. Unlike modalities containing directly identifiable features, endothelial images typically lack overt personal identifiers, which may reduce the necessity of federated architectures in certain contexts. At the same time, localization may be clinically meaningful. Endothelial morphology and disease presentation can vary according to ethnicity, genetic background, environmental exposure, diet, and regional clinical practices. Thus, while federated learning is an intriguing avenue for developing broadly applicable systems, further empirical research is required to determine whether globally trained models consistently outperform locally optimized systems tailored to specific populations and acquisition environments.

Importantly, simply increasing dataset size does not guarantee improved model performance—only larger datasets. Model robustness depends on data quality, representativeness, and appropriate training strategies. Consequently, complementary approaches such as self-supervised learning [55] and calibration techniques designed to address class imbalance [67] remain essential for developing reliable and clinically meaningful AI systems.

Self-supervised learning methods, which require few or no labels, offer an efficient way to leverage large datasets without the need for extensive manual annotation. This is particularly useful when dealing with ’unsure’ data in medical diagnoses, where certain cases cannot be definitively labeled due to lack of information or early-stage development [68]. Additionally, calibration techniques are vital in class-imbalanced scenarios, ensuring that deep learning models do not bias their predictions toward the majority class.

These approaches are essential, especially in AI’s clinical translation, where the variability of images and diseases must be accurately represented. They also play a crucial role in analyzing wider areas by stitching multiple images, a strategy recently validated in a pilot wide-field specular-imaging study [54], particularly useful in assessing the state of the endothelium in the presence of diseases like FECD.

### Robust image quality estimation

Despite significant advances in AI-based methods for specular microscopy, ensuring optimal image quality remains a formidable challenge from both hardware and software perspectives. The adage ’garbage in, garbage out’ is particularly relevant, as the quality of input images directly impacts the performance of AI models. Progress in specular microscope technology is necessary to capture high-quality images [69], whereas AI methods must concurrently develop mechanisms to assess and manage varying image quality levels within their processing pipelines.

Recent developments, such as the automatic region of interest (ROI) detection method by Vigueras et al. [50], demonstrate progress in addressing these challenges. Their Dense U-nets approach for identifying trustworthy areas in images, often plagued by blur, noise, and focus issues, represents a significant step in enhancing the reliability of AI-based image analysis. However, consistently obtaining high-quality images for precise analysis remains a challenge.

In addition to challenges in in vivo analysis, similar issues extend to ex vivo scenarios, such as the analysis of donor corneas. Kiefer et al. [70] proposed an AI-based Decision Support System to aid in the classification of Cornea guttata in post-mortem donor cornea images. This hybrid system underscores the potential of AI to improve image analysis in diverse contexts, yet also highlights the ongoing need for improved image quality and reliable AI interpretation.

The dual challenge of advancing imaging technology along with the development of robust AI algorithms for quality assessment underscores the complexity of achieving reliable analysis in corneal health assessment. While progress is being made, continuous efforts in both hardware improvement and algorithmic innovation are essential for overcoming these persistent challenges.

### Challenges in AI clinical translation

Translating AI systems from research to clinical practice in specular microscopy and similar medical imaging fields is a critical and urgent challenge. Despite the high performance of these systems in retrospective studies, their adoption in clinical settings remains limited [63]. Key obstacles include difficulties in generalizing AI systems across diverse clinical environments and navigating the ethical and legal complexities associated with medical data sharing [71].

Insights from related medical imaging fields highlight similar translation challenges. Achieving external validity, enhancing the interpretability of AI (“explainable AI”), and complying with regulatory requirements are common barriers that impede clinical integration [72–74]. Broader reviews of AI applications in corneal diseases report comparable limitations, including heterogeneous datasets, lack of standardized disease definitions, and insufficient prospective validation [75]. These issues are directly pertinent to corneal endothelium imaging, where adapting AI systems to established clinical practices and grading standards is essential.

The necessity of seamlessly integrating AI into existing clinical workflows is critical for its successful adoption, as discussed by Serag et al. [76]. Such integration must ensure that AI outputs are interpretable, reproducible, and aligned with clinician decision-making processes.

Moreover, the challenge lies not only in developing powerful AI systems but in ensuring that these systems are practical, reliable, and user-friendly for clinicians. Overcoming these barriers is essential for the routine clinical use of AI in specular microscopy and for advancing corneal health assessment and treatment planning.

### Reliability and reduced analyzed area

The reliability of corneal endothelium assessments in specular microscopy, particularly concerning the analyzed area, remains a significant concern. Despite advancements in AI models, providing accurate assessments from limited data obtained in small analyzed areas continues to be a challenge. This issue can be partially addressed by techniques such as acquiring multiple images of the same region to ensure a more comprehensive analysis (Fig 6).

Previous methods, such as those by Böhringer et al. [77] and Gasser et al. [78], focused on the variability of repeated images from the same eye and long-term tracking without employing AI-based robust segmentation. These approaches, while innovative, did not fully address the complexities in cases with FECD, which significantly challenge the analysis of specular microscopy images.

Recently, Prada et al. [26] introduced a method that computes a robust estimator (median) of morphometric parameters from multiple acquisitions. This technique effectively addresses the overestimation issues common in many built-in image analysis tools of specular microscopes and incorporates guttae characterization, providing a more comprehensive assessment of the corneal endothelium, especially in the presence of FECD. Considering wider areas by stitching multiple images together may be another viable approach to correctly assess the state of the endothelium, particularly under diseased conditions.

The continued exploration of techniques for analyzing both in vivo and ex vivo images, such as those of donor corneas, is crucial for enhancing the reliability of assessments in specular microscopy. The integration of AI-based robust segmentation methods in future work will be key in addressing the limitations of reduced analyzed areas and improving the accuracy of corneal health assessments.

These challenges highlight the complexities involved in the clinical adoption of AI tools in specular microscopy. For a more general overview of these challenges in ophthalomolgy, see Li et al. [38]. Addressing these issues is crucial for the successful integration of AI in clinical practice and the realization of its full potential in enhancing corneal health assessment.

## Conclusion

This review highlights significant advances in the application of artificial intelligence (AI) to specular microscopy for corneal endothelium analysis. AI-based methods have improved the accuracy and efficiency of segmentation and have addressed several limitations of traditional approaches, particularly in the presence of guttae.

Notably, recent work has enabled more faithful morphometric assessment in Fuchs endothelial corneal dystrophy, including indices such as effective endothelial cell density and guttae area ratio, which provide clinically relevant characterization beyond conventional density estimates and may be especially informative in early and intermediate disease stages.

Despite these advances, important challenges remain. Progress is increasingly constrained not by the choice of network architecture alone, but by dataset limitations and evaluation design: access to sufficiently large and clinically representative data, rigorous curation, reliable reference segmentations, and standardized acquisition and annotation protocols. In this context, training strategies such as self-supervised or semi-supervised learning, calibration methods, and robust image-quality assessment are central to improving generalizability and clinical reliability. Future work should therefore prioritize the development of reproducible data pipelines, transparent validation across diverse settings, and interpretable outputs aligned with clinical decision-making. With coordinated advances in these areas, AI-enabled specular microscopy can mature from research prototypes into practical tools that support corneal health assessment and the monitoring of emerging regenerative endothelial therapies.
